# The first iguanian lizard from the Mesozoic of Africa

**DOI:** 10.1098/rsos.160462

**Published:** 2016-09-21

**Authors:** Sebastián Apesteguía, Juan D. Daza, Tiago R. Simões, Jean Claude Rage

**Affiliations:** 1 CEBBAD (CONICET), Fundación de Historia Natural ‘Félix de Azara’, Universidad Maimónides, Hidalgo 775, 7°p (1405), Buenos Aires, Argentina; 2 Department of Biological Sciences, Sam Houston State University, 1900 Avenue I Lee Drain Building Suite 300, Huntsville, TX 77341-2116, USA; 3 Department of Biological Sciences, University of Alberta, Edmonton, Alberta, CanadaT6G2E9; 4 CR2P, Sorbonne Universités, UMR 7207 CNRS, CNRS, Muséum National d'Histoire Naturelle, Université Paris 6, CP 38, rue Cuvier, 75231, Paris cedex 05, France

**Keywords:** Acrodonta, biogeography, Cretaceous, Gondwana, phylogeny, Squamata

## Abstract

The fossil record shows that iguanian lizards were widely distributed during the Late Cretaceous. However, the biogeographic history and early evolution of one of its most diverse and peculiar clades (acrodontans) remain poorly known. Here, we present the first Mesozoic acrodontan from Africa, which also represents the oldest iguanian lizard from that continent. The new taxon comes from the Kem Kem Beds in Morocco (Cenomanian, Late Cretaceous) and is based on a partial lower jaw. The new taxon presents a number of features that are found only among acrodontan lizards and shares greatest similarities with uromastycines, specifically. In a combined evidence phylogenetic dataset comprehensive of all major acrodontan lineages using multiple tree inference methods (traditional and implied weighting maximum-parsimony, and Bayesian inference), we found support for the placement of the new species within uromastycines, along with *Gueragama sulamericana* (Late Cretaceous of Brazil). The new fossil supports the previously hypothesized widespread geographical distribution of acrodontans in Gondwana during the Mesozoic. Additionally, it provides the first fossil evidence of uromastycines in the Cretaceous, and the ancestry of acrodontan iguanians in Africa.

## 1. Introduction

The phylogenetic position of Iguania (chameleons, agamas, iguanas and New World lizards such as anoles) within Squamata is conflictive between morphological [1,2] and molecular or combined datasets [3–7], being recovered as a nearly diverging branch of the Squamata tree in the former, and nested with snakes and anguimorph lizards (monitors, gila monsters, glass lizards and alligator lizards) in the latter. However, there is a consensus agreement among these studies that acrodontans represent a monophyletic group composed by chameleons and agamas. Acrodontans represent a vast palaeotropical radiation, with extant species living in Africa, Asia, Oceania and parts of southern Europe [8–10]. However, the fossil record of non-acrodontan iguanians dates back to the Early Cretaceous [11] and diversifies into widely distant continental areas by the Late Cretaceous (e.g. [12–17]). The fossil record of acrodontans is considerably more limited.

The oldest putative record of acrodontans (*sensu* Conrad [1] and Gauthier *et al.* [2]) is from the Early–Middle Jurassic of India [18] (but see considerations by Jones *et al.* [19]). Later Mesozoic records come from the Early Cretaceous of China [20], Late Cretaceous of Brazil [21] and the Mid-Cretaceous of Myanmar [22]. Adding to this sparse early fossil record regarding the evolution of the group, phylogenetic and biogeographic studies have also come to conflicting conclusions regarding acrodontan biogeographic origins. Proposed centres of origin and/or dispersal of the crown clades include Eastern Laurasia [23], or Gondwana [18,24], or distinct centres in Eastern Gondwana and Eastern Laurasia [25]. Given that acrodontans alone comprise almost 40% of the extant iguanian species [10], a better understanding of the currently poor knowledge of early acrodontan evolution is paramount.

Africa is a key region of extant acrodontan biodiversity, thus being of major relevance to the study of acrodontan evolution. African acrodontans include large species assemblages of both agamids and chameleons that occupy diverse environments throughout the continent [9,10,25,26]. The oldest unquestionable published record of acrodontans in Africa is from the Oligocene of Egypt [27], with a potential record from the Palaeocene of Morocco [28], and unpublished acrodontans with possible uromastycine affinities from the Eocene of Algeria and Tunisia (J.C.R. and co-workers 2015, unpublished data). Therefore, the available fossil evidence suggests that African acrodontans reached that continent during the Cenozoic, possibly by means of dispersal from Asia [23,25].

Here, we describe an acrodontan fossil species from the early Late Cretaceous Kem Kem beds of Morocco, Northwest Africa. This is the earliest record of any iguanian from the entire Mesozoic of Africa, and brings the record of acrodontans in Africa to the Cretaceous. The new taxon provides valuable clues towards the evolution of acrodontans in Africa, and contributes to our knowledge of early acrodontan evolution worldwide.

## 2. Material and methods

### 2.1. Digital images

Photomicrography and measurements were done using a Nikon D800 36.3 MP digital camera (Tokyo, Japan) at the Muséum National d'Histoire Naturelle (MNHM) in Paris. The specimen is catalogued in the MNHN collection and is registered as collected by René Lavocat. Interpretative illustrations were traced on the photographs using Adobe® Illustrator® CC, 2014.1.1 Release.

### 2.2. Specimens used for comparison

*Agama agama* (FMNH 22190), *Trioceros jacksonii* (AMNH 99984, AMNH 84559), *Pogona barbata* (FMNH 51648)*, Physignathus cocincinus* (FMNH 255017), *Uromastyx acanthinura* (AMNH 71836, MCZ 27382), *Uromastyx aegyptia* (AMNH 73160, FMNH 78661), *Saara hardwickii* (UCA.5). The approximate position of the fossil in the skull was estimated using a CT scan of *Uromastyx aegyptia* available from http://digimorph.org.

### 2.3. Standard symbolic codes for institutional resource collections

Abbreviations used follow [29,30] and the following unlisted collection: UCA, University College Anatomy Collection and University of Central Arkansas, USA.

### 2.4. Anatomical nomenclature

Following [31–33], we term sub-dental shelf as the medial projection of the dentary bone that lies ventral to its teeth, and sub-dental ridge as the medial, usually thickened, margin of the sub-dental shelf [32]. In acrodontans (and most rhynchocephalians), the sub-dental shelf is commonly absent, but the dentary bears a ventrally projecting sub-dental ridge which can be quite deep and partially enclose the dorsal half of the Meckelian canal, as in *Uromastyx, Chamaeleo* and *Agama*.

### 2.5. Phylogenetic procedures

Character scores from the new taxon and *Gueragama sulamericana* [21] were added to a dataset comprised of morphological and molecular characters [34], and analysed under traditional maximum-parsimony (MP), implied weighting maximum-parsimony (IWMP), and Bayesian inference. Although a larger morphological dataset is available [25], it contains far less mandibular characters than [34], and only one character would have been scored for the new taxon described herein. The resulting dataset includes 30 taxa, with 22 mandibular morphological characters and 1968 molecular characters of mitochondrial data from ND2 and adjacent loci. Out of these, 544 molecular characters were identified as invariable sites (27% of total number of sites) and were removed for the MP analyses. Therefore, 22 morphological and 1424 molecular characters (total of 1446 characters) were analysed under MP. The MP analyses were run in TNT v. 1.1 64-bit (no taxon limit) [35] using 1000 RAS and tree search with TBR (no gains in the sampling of local optima were obtained using the New Technologies algorithms). IWMP is designed to downweight highly homoplastic characters [36,37] and was also used to analyse the dataset in TNT, using a *K* = 3.0. A third analysis using Bayesian inference was conducted in MrBayes v. 3.2.5 [38]. Morphological data were analysed under two distinct model parameters for morphological data: Mk + F81 + Γ and Mk + F81 + LN, whereas the molecular data were analysed under a GTR + I + Γ model (the best substitution model for this molecular dataset [34]). All remaining parameters were set with the default options of MrBayes. The number of generations was set to 1 000 000, with six chains and three swaps per cycle (sample frequency = 1000 and burn in fraction of 0.25). The Bayes factor based on harmonic means of the model likelihoods [39,40] was calculated in order to evaluate whether a gamma (Γ) or a lognormal (LN) distribution had a better fit to the morphological data. As recent studies indicate a LN distribution of rate variation might be preferred to the most widely used gamma distribution [41,42]. However, no significant differences were observed—2 log*_e_*(*B*_10_) < 2—and therefore only the analysis using a gamma distribution (default option) is reported herein.

In datasets with relatively small numbers of taxa (less than 50 taxa—such as the one herein), Bayesian inference has been consistently demonstrated to provide greater accuracy and efficiency in finding the correct trees when evolutionary rates vary among branches for molecular characters [43–45], which is a biologically sound assumption for both morphological and molecular characters [46–50]. More recently, it has also been demonstrated that Bayesian outperforms traditional parsimony for discrete morphological characters at variable evolutionary rates, with and without missing data [51,52], although with some potential loss of resolution [52]. Additionally, Bayesian analyses allow explicit considerations regarding variations in evolutionary rates and branch lengths among characters, and between the morphological and molecular partitions of the dataset [38,53–55]. For the above reasons, we illustrate the result of the Bayesian inference analysis as our preferred result (based on methodological reasoning). However, for comparative purposes, we also report on the results obtained from both the MP analyses (see also the electronic supplementary material).

## 3. Results

### 3.1. Systematic palaeontology

Squamata Oppel, 1811

Iguanomorpha Sukhanov, 1961

Iguania, Cope, 1864

Acrodonta, Cope, 1864

†*Jeddaherdan* gen. nov.

Type species †*Jeddaherdan aleadonta* sp. nov.

Figures 1 and 3.

**Figure 1.**
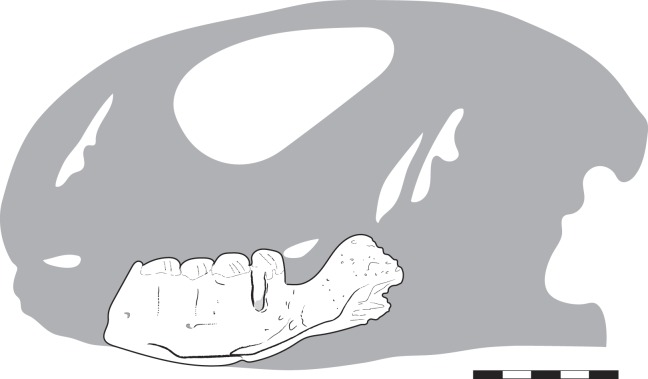
The estimated silhouette of the skull of MNHN.F.MRS51.1 is based on *Uromastyx aegyptia* (FMNH 78661). Scale bar equals 5 mm.

Included species †*Jeddaherdan aleadonta*

#### 3.1.1. Etymology

*Jeddaherdan* refers to its close relationships with *Uromastyx*, meaning in the Amazigh berber language from Morocco, grandfather (jeddi) of *Uromastyx* (aherdan); ‘aleadonta’, meaning ‘dice teeth’ in reference to the cube-like dentition.

#### 3.1.2. Holotype

MNHN.F.MRS51.1: isolated left partial mandible with teeth (figures 1 and 3).

#### 3.1.3. Type locality and stratigraphy

**Figure 2.**
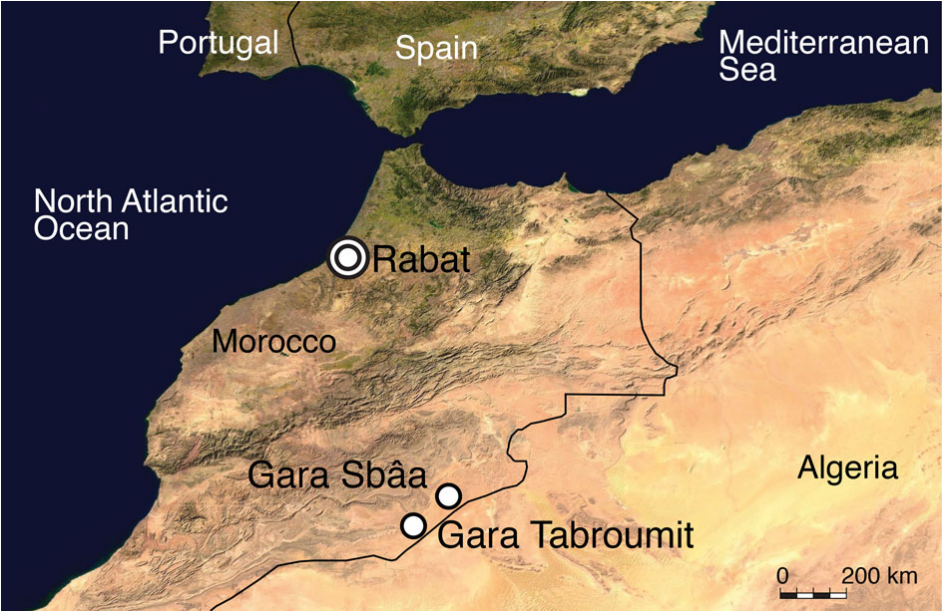
Position of the type locality (Gara Tabroumit) indicated on a satellite image of Northwest Africa.

Cenomanian (Late Cretaceous) beds from the Kem Kem region of Southeastern Morocco, Gara Tabroumit (figure 2). These deposits have superbly preserved fossils, including fishes, amphibians, crocodyliforms and dinosaurs [56–59].The expeditions carried on by René Lavocat (1909–2007) resulted in hundreds of specimens collected from 1948 to 1951, preserved in the collections of the MNHN. They belong to the three Kem Kem localities worked by Lavocat, (Gara Tabroumit, Kouah Trick and Gara Sbaa). The material described herein was among the fossils collected from the first locality, and had been originally misinterpreted as a fish jaw in the collection. No specific data for the age of Gara Tabroumit is available, so the age for the specimen can range between the base of the Cenomanian to the Early Turonian (*ca* 90–100.5 MYA) [59].

**Figure 3.**
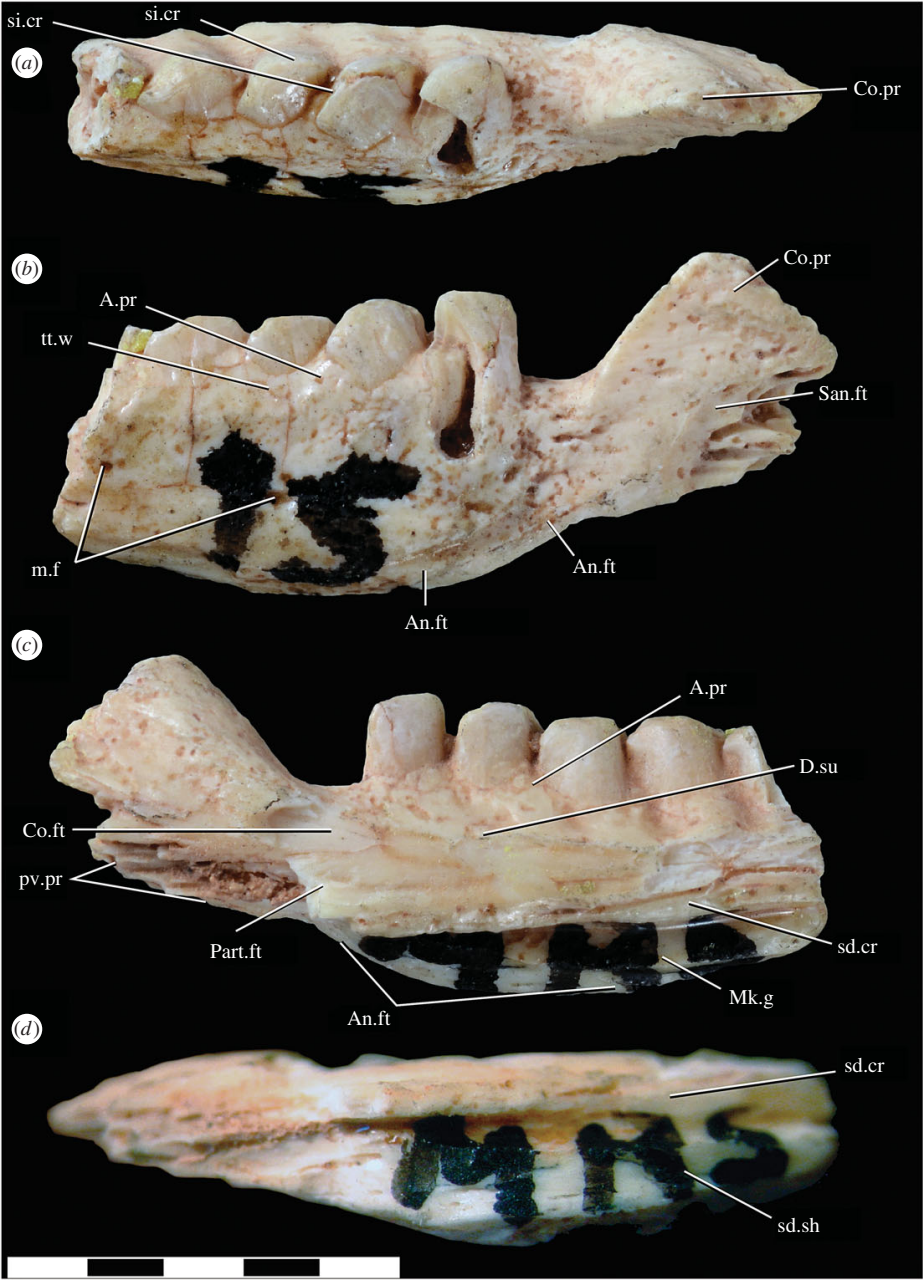
Holotype of *Jeddaherdan aleadonta* (MNHN.F.MRS51.1) in (*a*) dorsal; (*b*) lateral; (*c*) medial; (*d*) ventral views. Scale bar equals 5 mm. Abbreviations follow Evans [31]. An.ft, angular facet; A.pr, alveolar process; Co.ft, coronoid facet; Co.pr, coronoid process; D.su, dentary sulcus; m.f, mental foramen; Mk.g, Meckel's groove; pv.pr, posteroventral process; San.ft, surangular facet; sd.cr, sub-dental crest; sd.sh, sub-dental shelf; si.cr, sinusoidal crest; tt.w, tooth wear; Part.ft, prearticular facet.

#### 3.1.4. Diagnosis

*Jeddaherdan aleadonta* can be distinguished from other acrodontan lizards by the following combination of features: posterodorsally ascending coronoid process of dentary; sub-dental ridge projecting ventrally to mid-height of the Meckelian canal posteriorly; posterior dentary teeth very closely spaced, labiolingually compressed and mesiolabially oriented; dentary teeth with vertically straight mesial and distal margins, and almost flat apices, bearing mesiodistally oriented sinusoidal crests; the dentition also lacks any ornamentation (striations, flutings or facets) and bears an apical narrow groove extending mesiodistally.

#### 3.1.5. Description

The fossil is represented by an incomplete left dentary lacking the symphysial and posteriormost portions. The fossil includes the facets for articulation with the surangular, angular and coronoid bones. The dentary is a stout bone with a straight posterior ventral margin; it bears at least four teeth, and has a thick and well-developed posterodorsal extension of the coronoid process.

The teeth have long and wide tooth bases and look cubic in lateral view with a flat apical surface. In dorsal view, the contour of the teeth is parallelogramic, thus having an overall great similarity to the teeth of *Uromastyx* and *Saara* (uromastycines). Despite this the similarity, the relative size of the teeth is much greater than in extant uromastycines (by comparing with the depth of the dentary and size of the coronoid process). Therefore, the total number of dentary teeth is expected to be less than usually observed in extant uromastycines (between 14 and 17 in the adults of the observed specimens). Additionally, the consecutive teeth are interlocked, with the distolingual side of each tooth in parallel to the mesiolabial side of the tooth immediately posterior to it (providing a mesiolabially inclined configuration of the teeth). This interlocking is present for almost half the length of each tooth mesiodistal length, and defines an oblique and minimal interdental space. The teeth have a well-defined apical sinusoidal crest that is directed mesiolabially. Among extant acrodontan lizards, an apically positioned sinusoidal crest also occurs in uromastycines, but it is directed mesiolingually. The posterior dentition is apicolingually placed on the dentary labial wall, thus the dentary has a more prominent alveolar process on the labial rather than on the lingual side, and the tooth bases extend lingually to the dentary dorsal margin (a condition partially comparable in terms of tooth placement on the dentary to the terms ‘pleuroacrodont’ [18] or ‘sub-pleurodont’ [60]). The latter trait is seen in most agamids, whereas a strictly apically placed posterior dentition is most common (and perhaps exclusive) to chamaeleonids ([34] and T.R.S. 2015, personal observation). The posterior dentition is ankylosed to the dentary sub-dental shelf, and a comparatively reduced degree of fusion also occurs to the labial wall of the dentary. No resorption pits are visible, which indicates tooth replacement was absent, at least in the ontogenetic stage represented by this specimen (see below).

The labial surface of the teeth is inclined and has subtle furrows, which may be caused by diagenetic processes, whereas the lingual side is vertical and smooth. Wearing is evident on the alveolar processes on the labial side, marking where the lingual surface of the maxillary teeth occluded. According to the classification of teeth by Jones [61], the general morphology of the teeth in *J. aleadonta* is intermediate between the cut and slice, and between the grind and shred morphotypes, with a tooth shape similar to *Uromastyx* and *Saara*. This dental morphotype might be indicative of an omnivorous diet with an important herbivorous component, as indicated by the elongate mesiodistal axis of the teeth, and an apical sharp crest that could be used for shredding plant material. However, the lack of extensive tooth wear as that observed in old *Uromastyx* individuals suggests that the holotype of *J. aleadonta* represents a young specimen and/or that it fed on plant material much softer than that consumed by *Uromastyx*.

In the labial side of the dentary, there are at least two mental foramina. Also, on the labial side, the facets for the angular and surangular bones are preserved, with the angular facet extending anteriorly to the level of the midpoint of the penultimate tooth. The preserved posterior portion of the dentary has a medially open Meckel's canal. The ventral surface of the mandible is broken close to the base of the coronoid process, and at the anteriormost preserved region of the dentary. Therefore, it cannot be determined with precision if the posteroventral margin of the dentary curved inwards posteriorly (as in chamaeleonids and most agamines) in these regions. However, at the level of the angular facet of the dentary, the dentary is better preserved and the labial wall is vertically straight (as in leiolepidines). This suggests that, if any, the degree of inward inflection would be minor.

Attaching lingually to the sub-dental crest is a facet for the anteromedial process of the coronoid bone. This facet indicates that the coronoid abutted the sub-dental crest, right below a faint sulcus, or dental groove [62,63], that extends below the tooth bearing section of the dentary. The facet for the anteromedial process of the coronoid extends anteriorly to the level of the distal portion of the last dentary tooth, as also seen in taxa such as *Agama agama*. We discard the possibility of a large splenial in *J. aleadonta,* because there is no indication of a splenial facet on the sub-dental crest; therefore, the splenial in *J. aleadonta* was absent or reduced. Had this bone been present, it would have been positioned mostly ventral to the sub-dental crest, having a limited or no suturing contact with the latter structure, as is observed in many extant acrodontans (e.g. *Agama, Leiolepis* and *Uromastyx.*).

## 4. Discussion

### 4.1. Ontogenetic stage

In acrodontans, tooth replacement does not occur as observed in most other lizards. Some replacement occurs early during ontogeny in the anterior tooth series. However, in the posterior series, teeth are continuously added posteriorly to the back of the tooth row without replacement [64–67]. In the holotype of *J. aleadonta,* the observed degree of ankylosis of the posteriormost teeth to the sub-dental shelf, the amount of tooth wearing and the absence of teeth erupting posteriorly, leads to the conclusion that this specimen probably represents an individual in an advanced ontogenetic stage [66,67]. Nevertheless, the reduced degree of fusion of the posteriormost teeth to the labial wall compared to adults of extant acrodontans (and also *G. sulamericana*) raises the possibility that full skeletal maturity was still not attained.

### 4.2. Osteological comparison of *Jeddaherdan* with other lepidosaurs

*Jeddaherdan* shares with most rhynchocephalians and acrodontans some features such as lack of tooth replacement on the posterior tooth series (although replacement is observed in early rhynchocephalians, [68,69]), a coronoid process of the dentary that ascends posterodorsally, and a reduction or loss of the splenial [25,31,70]. However, *J. aleadonta* differs from all known rhynchocephalians and it is similar to most agamid acrodontans in having the following combination of features: apicolingually positioned posterior dentary teeth (tooth bases are placed apically, but also extend lingually to some degree), whereas they are placed entirely apically in most rhynchocephalians and all chamaeleonids; the presence of a very deep Meckel's groove; the absence of a secondary deposition of dentine along the tooth row; the presence of an angular bone that extends well anteriorly relative to the last tooth in medial aspect (revealed by the angular facet, which extends to the level of the anteriormost preserved tooth) and a dentary with a deep facet for the surangular [18,21,31].

*Jeddaherdan* shares some particularly informative features with extant acrodontans, such as the posterodorsally ascending coronoid process of the dentary, the squared-like shape of the posterior dentary teeth in lateral aspect and the almost horizontal apical tooth surfaces. The latter features are all common to *Uromastyx* and closely related taxa (uromastycines, e.g. AMNH 73160, FMNH 78661, MCZ 27382 and UCA.5), but uncommon to *Leiolepis* and other acrodontans (e.g. [25,31,71] and FMNH 22190, AMNH 99984, AMNH 84559, FMNH 51648, FMNH 255017). Among extant uromastycines, *Jeddaherdan* differs from *Uromastyx* and *Saara* by the mesiolabial orientation of its teeth (which are oblique, but mesiolingually oriented in *Uromastyx*), having a shallow sub-dental ridge, and a very elongate anteromedial process of the coronoid. The dorsolateral excavation of the dentary, producing a crest posterodorsally ascending towards the coronoid process, such as observed in *Uromastyx* and *Gueragama sulamericana*, is also absent in the new taxon. Furthermore, when compared with *Gueragama sulamericana*, the only other known acrodontan from the entire Cretaceous of Gondwana, *J. aleadonta* further differs from it in the form of tooth implantation (*J. aleadonta* has a much reduced lingual contribution of its tooth bases), absence of posterior tooth replacement, absence of a wide apically placed groove on the posterior tooth series, and by the presence of sinusoidal tooth apices.

There are marked differences between *Jeddaherdan* and the Palaeogene fossil acrodont *Barbaturex morrisoni* from Myanmar, which also bears uromastycine affinities [72]. Although both taxa have incomplete dentaries, *Barbaturex* has larger dentaries and an estimated SVL of about 1 m [72], whereas our size estimate for *Jeddaherdan* is no more than 10 cm in SVL (based on jaw/SVL ratio of *Uromastyx acanthinura* AMNH–R 71836, and assuming isometric scaling). Some additional morphological differences include: the lack of prominent ridges on the ventral surface of the dentary (very conspicuous in *Barbaturex*); the shape of the posterior dentary teeth, which have flat apical surfaces in *Jeddaherdan* versus triangular in *Barbaturex*; and consecutive posterior teeth arranged in an interlocking pattern in *Jeddaherdan,* instead of juxtaposed teeth as in *Barbaturex*. Additionally, the presence of an angular facet on the jaw of *Jeddaherdan* indicates that element was present, but not fused to the dentary as in *Barbaturex*. Finally, it is very likely that the splenial bone of *Jeddaherdan* was not as developed as in *Barbaturex* (see our inference about this bone above).

Additional important differences are also found between *Jeddaherdan* and other Palaeogene uromastycines, such as *Khaichinsaurus*, *Lentisaurus Lavatisaurus*, *Graminisaurus Agamimus* and *Acrodontopsis* from the Eocene of Mongolia [73], *Creberidentat* from the Eocene of China [74], and *Qianshanosaurus* from the Palaeocene of China [73,75], grouped under a clade termed Changjiangosauridae by Alifanov [73]. *Qianshanosaurus, Khaichinsaurus, Lentisaurus* and *Creberidentat* differ from *Jeddaherdan* by having considerably more teeth in the tooth row (at least 18, in *Qianshanosaurus,* 19 in *Creberidentat* and more than 20 in *Khaichinsaurus* and *Lentisaurus*); a very deep dentary posteriorly; a large splenial (in *Qianshanosaurus*), and labiolingually expanded tooth bases (in *Qianshanosaurus* and *Khaichinsaurus*, also possibly in *Creberidentat*) [73,75].

Some of the most similar Palaeogene taxa to *Jeddaherdan* include *Lavatisaurus*, *Graminisaurus Agamimus* and *Acrodontopsis* [73], an indeterminate uromastycine from the Eocene of Kyrgyzstan [60], *Uromastyx europaeus* from the Oligocene of France [76], and another indeterminate uromastycine from the Oligocene of Egypt [27]. All these taxa share with *Jeddaherdan* and most other uromastycines a posterodorsally ascending coronoid process of the dentary, teeth apicolingually placed on the dentary/maxillary labial margin, lower tooth counts (e.g. less than 14–17 as in extant *Uromastyx*), closely spaced tooth bases, teeth at least slightly elongated mesiodistally, and intensive apical tooth wear in most taxa. However, *Jeddaherdan* differs from all of these taxa by its very distinct interlocking and mesiolabially oriented teeth, and the teeth apical sinusoidal crest.

### 4.3. Phylogenetic position

**Figure 4.**
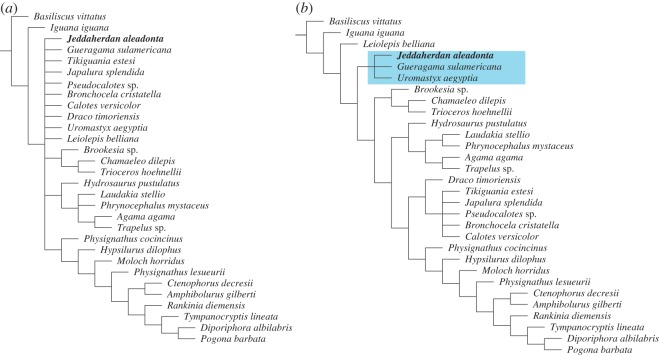
Maximum-parsimony analyses of the combined morphological and molecular datasets. (*a*) Strict consensus tree (8313 steps) inferred from 53 most parsimonious trees (7620 steps; consistency index = 0.357; retention index = 0.409 each) obtained from the traditional MP analysis. (*b*) Strict consensus (fit = 632.00542) of the five best fit trees (7631 steps, fit = 625.290572, CI = 0.357, RI = 0.408 in each tree) inferred from the MP analysis under implied weighting. Blue box = Uromastycinae.

*Jeddaherdan aleadonta* and *Gueragama sulamericana* are based on a single fragmentary dentary specimen each. Therefore, despite the relatively well-sampled amount of mandibular characters for acrodontans in the present dataset, the strict consensus of the traditional MP analysis could not find resolution at the early divergence of acrodontans (figure 4*a*). It can be determined, however, that *J. aleadonta* (and *G. sulamericana*) are nested within Acrodonta.

In the IWMP analysis (figure 4*b*), the early dichotomy between chamaeleonids and agamids, as obtained by Hutchinson *et al.* [34], is not retrieved, nor a monophyletic Leiolepidinae *sensu* Frost & Etheridge [77]. Instead, *Leiolepis* falls as the sister taxon to Uromastycinae (here being inclusive of *Uromastyx*, *Jeddaherdan* and *Gueragama*)*,* and a clade formed by Chamaeleontidae + Agaminae *sensu* Frost & Etheridge [77].

**Figure 5.**
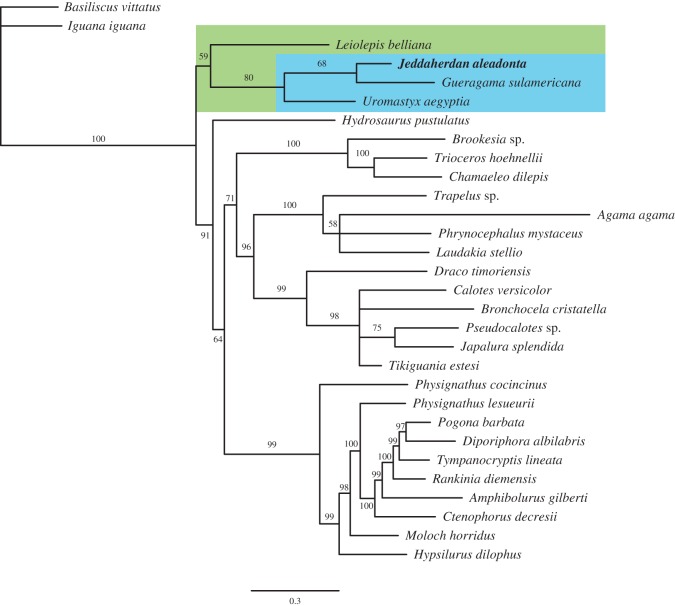
Bayesian consensus tree drawn from the 1073 trees obtained from the Bayesian inference of the combined datasets. Values on branches indicate clade posterior probabilities, and branches are proportional to their lengths. Green box = Leiolepidinae, blue box = Uromastycinae.

Under Bayesian inference (figure 5), the analysis we consider most appropriate to our dataset (see Material and methods), *Jeddaherdan* and *Gueragama* are nested with *Uromastyx*, as they also are in the IWMP tree (although forming a monophyletic Leiolepidinae with *Leiolepis*), and leiolepidines are the sister clade to Chamaeleontidae + ‘agamines’. The early divergence of leiolepidines (either forming a monophyletic group or a Hennigean comb), followed by the divergence of chamaeleontids and agamines, is a similar result to the one obtained using MP of morphological data by Gauthier *et al.* [2], and both ordered and unordered modified versions of that dataset in Simões *et al.* [78]. This overall topology was also obtained using molecular data under maximum-likelihood [4], and in combined dataset analyses of Wiens *et al.* [7] and Reeder *et al.* [3] using MP. All these results differ from another commonly inferred hypothesis of acrodontan relationships, in which the dichotomy between chamaeleontids and all other acrodontans (Agamidae) occurs early in acrodontan evolution, as previously inferred using both morphological and/or molecular data, under multiple tree inference methods [3,6,79–85], including the analysis of the present dataset without *Jeddaherdan* and *Gueragama* [34].

Importantly, under all of the phylogenetic analyses just discussed (and regardless of the position of chamaeleonids), the lineage leading to *Uromastyx* is always found as one of the earliest diverging ones in the phylogenetic history of acrodontans whenever *Uromastyx* or other uromastycines are included[1–4,6,7,79–85]. Therefore, it is implicit that the divergence of uromastycines might have been an ancient one in acrodontan evolution given our current knowledge of the clade. Considering the fossil evidence of acrodontans in the Cretaceous [20,21]—possibly even in the Jurassic [18]—and most molecular clock estimates for the divergence time for acrodontans [5,86–88] and uromastycines [19,85] (also estimated to be in the Cretaceous; but see [72]), the presence of Cretaceous uromastycines should not be unexpected. However, previous fossil findings only provided evidence for uromastycines, or closely related taxa, past the K-Pg boundary [27,60,72,75] with the oldest known uromastycines being from the Palaeocene of China [75]. *Jeddaherdan*, therefore, provides the first fossil evidence for the phylogenetically predicted presence of uromastycines in the Cretaceous.

The phylogenetic position inferred herein for *G. sulamericana*, previously inferred to lie at the stem of Acrodonta [21], is intriguing and of great relevance. *Gueragama* is nested within uromastycines along with *Jeddaherdan* (supported by features shared with uromastycines regarding the shape of the dentary bone). Different from *Jeddaherdan*, *G. sulamericana* has a very distinct dentition from other known uromastycines (including the presence of resorption pits), and has been inferred to represent plesiomorphic features of that taxon among other acrodontans [21]. The present tree topology (figure 5) indicates that the dentition and tooth replacement mode of *G. sulamericana* (similar to non-acrodontan iguanians), would be the result of a reversal within acrodontans and not a plesiomorphic trait. Nevertheless, the result for *G. sulamericana* could be affected by the reduced number of non-acrodontan iguanians in the dataset used herein, and thus increasing the sampling of the latter could help to better assess the transition between acrodontans and other iguanians. A future updated morphological dataset of acrodontan iguanians containing a diverse sample of well-constructed morphological characters (and with a more complete sampling of non-acrodontan iguanians) shall provide important insights into early acrodontan evolution, and the position of *Gueragama* within the group.

One important consideration in every phylogenetic analysis of very fragmentary fossil taxa is the potential effects of missing data upon their placement on the tree, which may cause fossils to be found more rootward on the tree [89–91]. However, as some studies have previously indicated, even very incomplete fossils can be accurately placed if they preserve key synapomorphies that allow their correct systematic placement by phylogenetic inference methods [92,93]. In the case of *Jeddaherdan* and *Gueragama* in the analyses herein, the potential rootward slippage caused by missing data does not seem to be an issue. Both are found in a crown group (uromastycines) along with an extant taxon (*Uromastyx*), and do not lie at the leiolepidines or acrodontan stem. This might be a consequence of the unique set of traits on the shape of the dentary that are quite conspicuous to uromastycines, and sometimes also in *Leiolepis* (characters 9 and 12 herein), and their tooth shape too (character 4 herein), which is quite different from the condition observed in other acrodontan lineages.

### 4.4. Biogeographic implications

The presence of a second acrodontan in West Gondwana during the Late Cretaceous provides important clues towards the early evolution of the group. *Jeddaherdan* represents the earliest iguanian known from Africa, and one of the few known acrodontans *sensu strictu* from anywhere in the world during the Cretaceous (considering that priscagamids have most recently been proposed to be the sister taxon to acrodontans, and not within the latter [1–3]). The acrodontans *Xianglong* from the Early Cretaceous of China [20] and *Bharatagama* from the Early–Middle Jurassic of India [18] represent the oldest members of the group (but see considerations concerning *Bharatagama* in [19]). *Jeddaherdan* thus represents a biogeographic link between the Asian Cretaceous forms and the recently discovered South American record (*Gueragama*).

Uromastycines and other agamids are currently widespread in northern Africa [8,26,94], with at least 11 uromastycine species endemic to that region ranging from the Atlantic coast of the Sahara Desert to the western margin of the Red Sea [10]. Previous records indicated the presence of acrodontans with affinities to uromastycines in the Oligocene of Egypt [27] and Eocene of Algeria and Tunisia (see above), but no record was known from the Cretaceous. Therefore, *Jeddaherdan* indicates uromastycines occurred in Africa at least as far back as the early Late Cretaceous, about 40–45 million years before the currently known Cenozoic records. Additionally, *Jeddaherdan* fills a biogeographical gap and confirms previous predictions of the widespread distribution of acrodontan iguanians (along with other iguanians) during the Cretaceous [21]. Given our phylogenetic hypothesis presented above (using our preferred topology, from Bayesian inference—see Material and methods), the oldest record of uromastycines, and of acrodontans in western Gondwana, would be close to the time of the final separation between South America and Africa (Albian-Cenomanian) [95,96]. Whether the distribution of Mesozoic acrodontans on both sides of the early Atlantic Ocean represents a dispersal event or the result of vicariance is currently unknown due to the paucity of fossil data.

Most of the currently known terrestrial limbed lizards from the Aptian/Albian to the Maastrichtian (‘middle’ to Late Cretaceous) of South America come from lower latitudes when compared with similar aged rhynchocephalians [21], which, in turn, were incredibly more abundant than terrestrial lizards in higher latitudes [97,98] in a ratio of 200 : 1. The discovery of *Jeddaherdan* indicates lizards also inhabited lower latitudes during the Late Cretaceous of Africa, where no sphenodontians are currently known from. Furthermore, the Cenomanian fossil record shows abundant herbivorous rhynchocephalians southwards from the Caiuá Desert in South America [97] and the presence of at least partially herbivorous lizards and no rhynchocephalians in northern Africa. If eastern South American and western African faunas had a similar terrestrial lepidosaurian composition during and immediately after the break-up of West Gondwana, then it is possible that further corresponding patterns of faunal distribution between Africa and South America may be revealed with the discovery of additional African material.

## Supplementary Material

The Supplementary Material File contains phylogenetic dataset scoring for the new taxon and additional information from our results.

## Data accessibility

The datasets supporting this article have been uploaded as part of the supplementary material. They are also available from the Dryad Digital Repository [99] http://dx.doi.org/10.5061/dryad.pn35j.
