# Supplementary material for: The first iguanian lizard from the Mesozoic of Africa
Source: R Soc Open Sci. 2016 Sep 21;3(9):160462. doi: 10.1098/rsos.160462 (PMC5043327; doi:10.1098/rsos.160462)
Supplement: The Supplementary Material File contains phylogenetic dataset scoring for the new taxon and additional information from our results. [file rsos160462supp1.docx]

**Electronic Supplementary Material**

Sebastían Apesteguía, Juan D. Daza, Tiago R. Simões, and Jean Claude Rage

**1. Retrieved unambiguous morphological synapomorphies**

Synapomorphies for Acrodonta from the strict consensus tree from the combined dataset under traditional TBR:

Acrodonta: Char. 3: 0 🡪 1; Char. 4: 0 🡪1; Char. 5: 0 🡪1; Char. 8: 0 🡪1; Char. 10: 0 🡪1; Char. 11: 0 🡪1; Char. 15: 0 🡪1; Char. 16: 0 🡪1; Char. 17: 0 🡪1; Char. 19: 0 🡪1; Char. 20: 1 🡪2

Synapomorphies leading to uromastycines from the strict consensus tree from the combined dataset under TBR with implied weighting:

Acrodonta: Char. 5: 0 🡪 1; Char. 11: 0 🡪 1; Char. 14: 0 🡪 1

Acrodonta (–*Leiolepis*): Char. 1: 0 🡪 2; Char. 10: 0 🡪 1

Uromastycinae: Char. 12: 0 🡪 1

Synapomorphies leading to uromastycines from the consensus tree from the combined dataset under Bayesian inference:

Leiolepidinae: Char. 9: 0 🡪 1

Uromastycinae: Char. 10: 0 🡪 1; Char. 12: 0 🡪 1

**2. Scorings for the morphological characters of *Jeddaherdan aleadonta* and *Gueragama sulamericana* in the dataset used herein.**

*Jeddaherdan aleadonta 2??010??1??101-???????*

*Gueragama sulamericana 2000000011?101-???????*

* *Iguana iguana* wasscored as “?” for morphological character 9 (ventral margin of dentary), which we re-scored as “0” based on personally observed specimens (Carnegie Museum of Natural History: CM (114409 and 35157).
